# Association of fibrinogen and plasmin inhibitor, but not coagulation factor XIII gene polymorphisms with coronary artery disease

**DOI:** 10.5937/jomb0-26839

**Published:** 2021-03-12

**Authors:** Ana Bronić, Goran Ferenčak, Robert Bernat, Jasna Leniček-Krleža, Jerka Dumić, Sanja Dabelić

**Affiliations:** 1 Sestre Milosrdnice University Hospital Centre, Clinical Institute of Chemistry, Department for Laboratory Diagnostics in Traumatology and Orthopaedics, Zagreb, Croatia; 2 Medicol Outpatients Clinic, Department of Laboratory Diagnostics, Zagreb, Croatia; 3 Westpfalz-Klinikum GmbH, Department of Internal Medicine 2, Kaiserslautern, Germany; 4 Children's Hospital Zagreb, Department of Laboratory Diagnostics, Zagreb, Croatia; 5 University of Zagreb, Faculty of Pharmacy and Biochemistry, Department of Biochemistry and Molecular Biology, Zagreb, Croatia

**Keywords:** coronary artery disease, FXIII, fibrinogen, genotyping, plasmin inhibitor, polymorphisms, koronarna arterijska bolest, FXIII, fibrinogen, istraživanje genotipa, inhibitor plazmina, polimorfizmi

## Abstract

**Background:**

In the final phase of clot formation, fibrinogen constitutes frame, whereas factor XIII (FXIII) active form is responsible for the covalent cross-linking of fibrin fibres and plasmin inhibitor (PI), thus contributing to clot stability. It could be expected that any change of coagulation factors' structure affects the clot formation and modulates the atherothrombotic risk. The aim was to determine the frequency of four single nucleotide polymorphisms: (*i*) A > G in codon 312 of the fibrinogen α-chain gene (rs6050, Thr312AlaFGA), (*ii*) C > T at position 10034 of the 3 - untranslated region in the fibrinogen γ-chain gene (rs2066865, 10034C > T FGG), (*iii*) C > T in codon 564 of the FXIII-A subunit gene (rs5982, Pro564LeuFXIII-A), and (*iv*) C > T in codon 6 of the plasmin inhibitor gene (rs2070863, Arg6TrpPI) in Croatian patients and their association with coronary artery disease (CAD).

**Methods:**

We performed the unrelated case-control association study on the consecutive sample of patients 18 years old, who had undergone coronary angiography for investigation of chest pain and suspected CAD. The cases were patients with confirmed CAD (N=201), and the controls were the subjects with no CAD (N=119). Samples were genotyped using PCR-RFLP analysis.

**Results:**

Observed frequencies of the rare alleles of Thr312Ala FGA, 10034C > T FGG, Leu564Pro FXIII-A and Arg6Trp PI polymorphisms were 21%, 17%, 14%, 20%, respectively. Patients with 10034C > T FGG CC genotype had 3.5 times (95% CI 1.02-12.03) higher adjusted odds for CAD than patients with 10034C > T FGG TT genotype. Patients with Arg6Trp PI CC genotype had 3.86 times (95% CI 1.23-12.12) higher odds for CAD than patients with Arg6Trp PI TT genotype. It seems that those genotype-related higher odds are also male-gender related. No difference was observed regarding any other investigated polymorphism.

**Conclusions:**

Our finding suggests that 10034C > T FGG and Arg6Trp PI are associated with CAD.

## Abstract (sr)

**Uvod:**

U završnoj fazi stvaranja ugruška fibrinogen čini okvir, dok je aktivni oblik faktora XIII (FXIII) odgovoran za kovalentno umrežavanje vlakana fibrina i inhibitora plazmina (PI), doprinoseći tako stabilnosti ugruška. Moglo bi se očekivati da svaka promena strukture faktora koagulacije utiče na stvaranje ugruška i modulira rizik od aterotrom - bota. Cilj je bio da se odredi frekvencija četiri pojedinačna nukleotidna polimorfizma: (I) A > G u kodonu 312 gena a-lanca fibrinogena (rs6050, Thr312AlaFGA), (II) C > T na položaju 10034 3' netransliranog regiona u genu g-lanca fibrinogena (rs2066865, 10034C > TFGG), (III) C > T u kodonu 564 gena podjedinice FXIII-A (rs5982, Pro564LeuFXIII-A), i (IV) C > T u kodonu 6 gena inhibitora plazmina (rs2070863, Arg6TrpPI) kod hrvatskih pacijenata, i njihovu povezanost sa koronarnom arterijskom bolešću (CAD).

**Metode:**

Obavljeno je nepovezano istraživanje slučajeva i kontrola na uzastopnom uzorku pacijenata sa 18 i više godina koji su bili podvrgnuti koronarnoj angiografiji radi ispitivanja bola u grudima i sumnje na CAD. Slučajevi su bili pacijenti sa potvrđenom CAD (N = 201), a kontrolna grupa su bili oni koji nisu imali CAD (N = 119). Genetska konstrukcija uzoraka istražena je pomoću PCR-RFLP analize.

**Rezultati:**

Posmatrane frekvencije retkih alela Thr312Ala FGA, 10034 C > T FGG, Leu564Pro FXIII-A i Arg6Trp PI polimorfizma bile su, redom, 21%, 17%, 14%, 20%. Pacijenti sa 10034 C > T FGG CC genotipom imali su 3,5 puta (95% CI 1,02-12,03) veće prilagođene izglede za dobijanje CAD u odnosu na pacijente sa 10034 C > T FGG TT genotipom. Pacijenti sa Arg6Trp PI CC genotipom imali su 3,86 puta (95% CI 1,23-12,12) veće izglede za CAD u odnosu na pacijente sa Arg6Trp PI TT genotipom. čini se da ti veći izgledi vezani za genotip takođe imaju veze i sa muškim polom. Nije uočena razlika u odnosu na bilo koji drugi istraženi polimorfizam.

**Zaključak:**

Dobijeni rezultati ukazuju na to da su 10034 C > T FGG i Arg6Trp PI povezani sa CAD.

## Introduction

**Figure 1:**
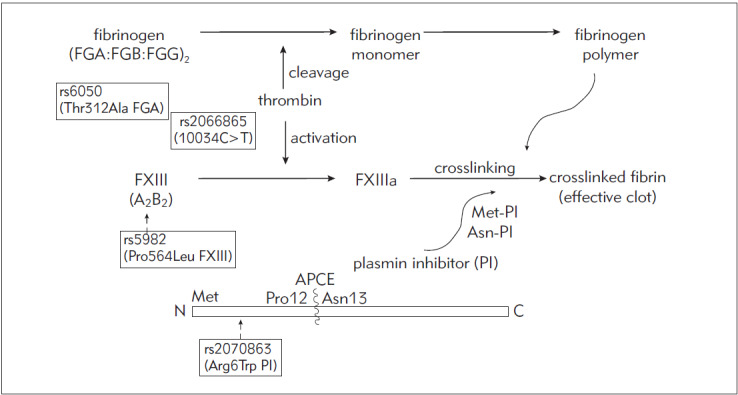
Schematic diagram of a part of the coagulation cascade with indicated SNPs of interest. Upon thrombin-induced cleavage of a hexameric molecule of fibrinogen, comprised of a duplicate set of FGA, FGB and FGG subunits, fibrin polymers are produced. Coagulation factor FXIII, composed of two catalytic A and two carrier B subunits, is activated into FXIIIa by thrombin. FXIIIa catalyses cross-linking of fibrin polymers and plasmin inhibitor (PI) into an effective clot. Antiplasmin cleaving enzyme (APCE) cleaves Met-PI into Asn-PI, which rate of cross-linking is different from its precursor Met-PI.

Primary causes of death in developed countries are cardiovascular diseases, among which coronary artery disease (CAD) is the most predominant and fatal. Besides the traditional risk factors, which are well-defined (family history, age, gender, diabetes, cigarette smoking, hypertension, hypercholesterolemia, low HDL cholesterol, hypertriglyceridemia, and obesity), various genetic variants (so far known to a much lesser extent) can also represent determinants that influence the individual susceptibility for CAD [1] [2] [3] [4]. CAD is a disease in which stenosis or clot in the coronary blood vessels leads to spectra of clinical phenotypes, among which myocardial infarction (MI) is one of the most dangerous [5] [6], and atherosclerosis or arteriosclerosis is the primary cause of CAD. Clot formation is a strictly controlled process and depends on the complex interaction between the individual components of the haemostasis system. Any imbalance in the system may affect the clot structure, stability and its resistance to fibrinolysis. Thus, all the elements involved in the clot formation are important determinants of atherothrombotic risk, and so are fibrinogen and coagulation factor XIII (FXIII) [7] [8] [9] [10]. Fibri(noge)n constitutes cloth frame, whereas the activated form of FXIII (FXIIIa) is responsible for the mechanical strength and cloth stabilisation by covalent cross-linking of fibrin fibres and incorporation of plasmin inhibitor (PI, also known as α2-antiplasmin), into the cloth [11] [12] [13] [14] [15] (Figure 1).

Results published so far implicate a potential association of particular polymorphisms in genes coding factors important for clot stability with clot structure.

Single nucleotide polymorphisms (SNP)db SNP cluster IDrs6050 and rs2066865 in fibrinogen genes are, among other two, in the focus of this study. Three fibrinogen genes code for alpha (α, FGA), beta (β, FGB) and gamma (γ, FGG) chains, and duplicate sets of those chains comprise the hexameric molecule of fibrinogen. dbSNP rs6050 causes the substitution of threonine by alanine (Thr312Ala FGA). Thr312Ala FGA is located within positions 242-424 in Ca fibrinogen domain, a location important for the lateral aggregation and processes dependent on FXIIIa [16]. Moreover, dbSNPrs2066865 is 10034 C > T substitution in the 3'-untranslated region of the FGG gene. Allele C favours the formation of fibrinogen γ'-chain instead of alternatively spliced γ'-chain, which is less commonly present (in usually 8%-15% of plasma fibrinogen [17]. Therefore, both SNPs can affect clot overall density and stability.

SNP Pro564Leu FXIII-A, a result of C > T substitution (dbSNP cluster ID rs5982), is located in the catalytic subunit A of a coagulation factor FXIII, which is a dimer of two catalytic subunits (FXIII-A) and two carrier subunits (FXIII-B). The 564Leu variant is known to lower FXIII plasma level and increase FXIII activity [18].

The SNP C > T (dbSNP cluster ID rs2070863) within codon 6, exon 3 of the plasmin inhibitor gene (SERPINF2) results in Arg6Trp substitution in PI. That SNP affects the rates of APCE (antiplasmin cleaving enzyme) cleavage of PI at the position 12-13 (Pro12-Asn13), and the ratio of the subsequent product, Asn-PI, and the precursor Met-PI. In human plasma, approximately 30% of PI circulates as uncleaved Met-PI and ~70% as Asn-PI. Asn-PI can be cross-linked into fibrin 13 times faster than its precursor, and the plasma clot lyses time is increased inversely to the Met-PI/Asn-PI ratio [19].

Taking into consideration that the prevalence of the particular allele varies among populations and that polymorphisms can have a different impact in various environments, our aim was to estimate the frequency of genotypes and alleles of candidate SNPs: Pro564LeuFXIII-A, Thr312AlaFGA, 10034 C > TFGG, Arg6TrpPI and their possible association with coronary artery disease and myocardial infarction in Croatian patients who underwent coronary angiography for investigation of chest pain and suspected CAD.

## Materials and Methods

### Study design, setting and population

We performed the unrelated case-control association study on samples of overall 320 patients of Magdalena Special Hospital for Cardiovascular Surgery and Cardiology in Croatia. The study was performed according to the Declaration of Helsinki and was approved by the Ethics Committees of the Magdalena Special Hospital (No. 195/Odl.-2211/10 from May 25 2010) and the University of Zagreb Faculty of Pharmacy and Biochemistry (No. 643-03/11-01/4 from January 14, 2021). All patients signed the informed consent for participation.

According to the inclusion criteria, the targeted population were patients of both genders ≥ 18 years old, who had undergone coronary angiography for investigation of chest pain and suspected CAD, regardless of the previous history of MI. The disease severity was evaluated by counting the number of major epicardial coronary arteries affected by significant stenosis (≥ 50%). Exclusion criteria were a history of coronary artery bypass surgery, previous stenting procedure or incomplete angiogram scoring. Inclusion criteria for the control group were angiographically documented normal coronary arteries, while exclusion criteria were stenosis in angiogram (subjects with more than 10% stenosis of major coronary arteries), history of atherosclerosis, or evidence of atherosclerosis in other vascular beds. At the time of blood sampling, a complete clinical and pharmacological history, including the presence or absence of cardiovascular risk factors such as smoking, hypertension, and diabetes mellitus, was obtained from the patients. Previous myocardial infarction was defined on the basis of medical records/documentation on hospitalisation or ECG changes and the presence of elevated biochemical markers of myocardial necrosis. This analysis was conducted on the part of the same study population on which we examined the association of Val34LeuFXIII polymorphism and CAD. We reported the diagnostic criteria for particular diseases and conditions and blood sampling in Bronic et al [20]. The total study population was divided into two major groups (CAD+ were the patients with confirmed CAD and CAD-the patients with no CAD). We selected a consecutive sample of patients in the order of their arrival for the coronary angiography exam. Additional subgrouping of CAD patients was performed based on the history of MI.

### DNA extraction and genotyping

All material was purchased from Roche Diagnostics, Manheim, Germany, if not stated otherwise. The isolation of genomic DNA from the whole blood containing EDTA as anticoagulant was performed by standard salting-out method [21]. Four SNPs in different genes were chosen for the study: (*i*) A > G in codon 312 of the FGA gene (db SNP cluster ID rs6050, Thr312Ala FGA), (*ii*) 10034 C > T substitution in the 3'-untranslated region of the FGG gene (db SNP cluster ID rs2066865), (*iii*) C > T in codon 564 of the FXIII-A subunit gene (db SNP cluster ID rs5982, Pro564Leu FXIII-A) and (*iv*) C > T in codon 6 of the PI gene (db SNP cluster ID rs2070863, Arg6Trp PI). The PCR-RFLP analysis was used for genotyping. The sequences of the newly designed primers were as follows: 5'-TATGGTTTTTGTTTTGTTAT-3' (forward FGG), 5'-CAGCCTGGCCAACATAGTGAAAT-3' (reverse FGG), 5'-CCGCAGATGAGAATGGAGTTAGAG-3' (forward FGA), 5'-CAGATTCAGAGTGCCATTGTCCAG-3' (reverse FGA), 5'-TCCCCCAGGT-CAAGAAGAAGAGA-3' (forward FXIII-A), 5'-CAGCCTGGCCAACATAGTGAAAT-3' (reverse FXIII-A), 5'-GCTCTGGGGGCTCCTGGTGCTCA-3' (forward PI), 5'-AGCAGAGACCCCACCCCAGAGCAG-3' (reverse PI). The final amplification mixture contained 4 mL of genomic DNA, 0.5 μmol/L of each primer, 200 μmol/L dNTPs, 1x PCR buffer (50 mmol/L KCl, 10 mmol/L Tris/HCl, pH 8.3), 1.5 mmol/L MgCl 2 and 0.6 U FastStart Taq polymerase. Each mixture was submitted to the following cycling conditions: one cycle of initial denaturation at 95°C for 5 minutes followed by 35 alternating cycles of denaturation (95°C, 1 min), annealing (65°C, 30 sec for FGA; 57°C, 1 min for FGG; 65°C, 1 min for FXIII-A; 70°C, 1 min for PI) and elongation (72°C, 1 min) as well as a single final elongation step at 72°C for 7 minutes. The effectiveness and specificity of amplification were confirmed by 1.5% agarose electrophoresis. The PCR products of FGA, FGG, FXIII-A and PI were digested for 3 hours at 37°C using 0.5 U/10 μL of restriction enzymes Rsal, KpnI, AcuI and HpaII, respectively.

Fragments were analysed using 2.5% agarose electrophoresis in TAE buffer and visualised at wavelength 254 nm on transilluminator with SyberSafe (Thermo Fischer). An additional step was included following amplification and digestion of the FGA gene fragment in order to detect the exact sequence and presence of AG or GG by sequencing samples with the presence of 117 bp fragments in commercial service Macrogen (Seoul, Korea). Three samples with known genotype were added in each analysis, and the control sample genotyping confirmed the reproducibility of the procedures.

### Statistical analysis

In order to determine if the genotypes are in Hardy-Weinberg equilibrium, we used the Chi-square (χ^2^) test, with equilibrium considered at p > 0.05. The Mann-Whitney U test was performed to investigate the association between fibrinogen levels and genotype. To investigate the association between the CAD and MI risk and certain genotypes, we used a multivariable binary logistic regression and presented odds ratios (OR) and their 95% confidence intervals (CI). We included data on age, gender, body mass index, current smoking, total cholesterol, HDL-cholesterol and triglycerides as covariates to calculate adjusted OR. We performed the classification and analysis of four polymorphisms interactions using the classification and regression tree analysis (CART) with the cross-validation performed using 25 subsamples. We set the level of statistical significance at two-tailed p<0.05, and confidence intervals at 95%. In the analysis of the secondary outcome, we corrected the p-values for multiple testing, using the sequential Holm-Bonferroni method. We performed a statistical analysis using the R free software [22].

## Results

We enrolled 320 Croatian inhabitants, 201 (62.8%) of them with confirmed CAD. Among them, 103/201 (51.2%) had a history of MI. The patients with CAD were somewhat older, more often men, with a higher prevalence of hypertriglyceridemia, smoking, diabetes mellitus and arterial hypertension. The CAD patients with a history of MI were more often men, with a higher prevalence of obesity, smoking and diabetes mellitus, but the prevalence of arterial hypertension was higher in the CAD patients with no history of MI. The women enrolled in this study were somewhat older than men, and a smaller percentage of them were smokers. It can be noticed that among the female subjects with CAD, overall triglyceride, and cholesterol levels were a little higher, and this group also comprised more subjects with diabetes and hypertension (Table 1).

**Table 1:** Participant demographic and clinical characteristics, shown for all individuals (T), male (M) and female (F). Data are presented as median (IQR) if not stated otherwise. CAD, coronary artery disease; MI, myocardial infarction; IQR, interquartile range; BMI, body mass index calculated as kg/m^2^; ERF, established risk factors (total cholesterol/HDL cholesterol ratio).

	CAD+ (n T=201) (n M=140) (n F=61)	CAD- (n T=119) (n M=59) (n F= 60)	CAD+ group only	
			MI+ (n T=103) (n M=76) (n F=27)	MI- (n T=98) (n M=64) (n F=34)
Age (years)	T 63 (55-69) M 61 (54-68) F 65 (60-71)	T 60 (52-66) M 56 (48-65) F 62 (55-66)	T 62 (54-70) M 59 (53-69) F 65 (62-71)	T 63 (56-69) M 63 (54-69) F 64 (59-69)
Men, n (%)	T 140 (69.7)	T 59 (49.6)	T 76 (73.8)	T 64 (65.3)
BMI (kg/m^2^)	T 28 (25-30) M 28 (25-31) F 27 (25-31)	T 28 (25-30) M 28 (25-30) F 28 (25-31)	T 28 (25-31) M 28 (26-31) F 29 (24-31)	T 27 (25-30) M 27 (25-30) F 27 (25-30)
Obesity (BMI ≥ 30.0), n (%)	T 60 (29.9) M 42 (30.0) F 18 (29.5)	T 33 (27.7) M 12 (20.3) F 21 (35.0)	T 33 (32.0) M 23 (30.3) F 10 (37.0)	T 27 (27.6) M 19 (29.7) F 8 (23.5)
Total cholesterol (mmol/L)	T 5.6 (4.7-6.4) M 5.3 (4.6-6.2) F 6.10 (5.20-6.80)	T 5.6 (5.0-6.4) M 5.4 (5.0-6.3) F 5.8 (5.2-6.6)	T 5.3 (4.5-6.0) M 5.3 (4.5-5.9) F 5.7 (4.7-6.2)	T 5.9 (5.0-6.8) M 5.4 (4.8-6.7) F 6.4 (5.5-7.3)
Elevated total cholesterol (> 5.0 mmol/L)	T 133 (66.2) M 86 (61.4) F 47 (77.1)	T 89 (74.8) M 41 (69.5) F 48 (80.0)	T 62 (60.2) M 43 (56.6) F 19 (70.4)	T 71 (72.4) M 43 (67.2) F 28 (82.4)
HDL cholesterol (mmol/L)	T 1.1 (0.9-1.3) M 1.0 (0.9-1.2) F 1.2 (1.0-1.4)	T 1.3 (1.1-1.5) M 1.2 (1.0-1.3) F 1.3 (1.2-1.5)	T 1.0 (0.9-1.3) M 1.0 (0.8-1.2) F 1.1 (0.9-1.3)	T 1.1 (1.0-1.3) M 1.1 (0.9-1.2) F 1.3 (1.1-1.9)
Lowered HDL cholesterol (< 1.0 mmol/L)	T 54 (26.9) M 44 (31.4) F 10 (16.7)	T 12 (10.1) M 7 (11.9) F 5 (8.2)	T 36 (35.0) M 28 (36.8) F 8 (29.6)	T 18 (18.4) M 16 (25.0) F 2 (5.9.)
ERF ratio	T 5.1 (4.2-6.1) M 5.2 (4.2-6.1) F 4.9 (4.1-6.1)	T 4.5 (3.8-5.1) M 4.7 (3.9-5.4) F 4.3 (3.6-4.9)	T 5.1 (4.2-6.1) M 5.3 (4.2-6.1) F 4.8 (4.1-6.0)	T 5.1 (4.2-6.0) M 5.1 (4.2-6.0) F 5.0 (3.8-6.2)
Triglycerides (mmol/L)	T 1.8 (1.3-2.6) M 1.7 (1.2-2.5) F 2.9 (2.40-4.0)	T 1.5 (1.1-2.2) M 1.4 (1.1-2.1) F 1.6 (1.2-2.4)	T 1.8 (1.3-2.7) M 1.8 (1.3-2.7) F 2.1 (1.5-2.6)	T 1.8 (1.3-2.6) M 1.7 (1.2-2.4) F 1.9 (1.5-2.4)
Hypertriglyceridemia (> 1.7 mmol/L), n (%)	T 111 (55.2) M 71 (50.7) F 40 (65.6)	T 49 (41.2) M 22 (37.3) F 27 (45.0)	57 (55.3) M 39 (51.3) F 18 (66.7)	54 (55.1) M 32 (50.0) F 22 (55.0)
Fibrinogen (g/L)	T 2.80 (2.30-3.65) M 2.8 (2.2-3.5) F 2.9 (2.4-4.0)	T 2.60 (2.10-3.40) M 2.4 (2.0-2.9) F 3.1 (2.3-3.7)	T 2.80 (2.30-3.50) M 2.8 (2.3-3.7) F 2.8 (2.4-3.5)	T 2.90 (2.30-3.70) M 2.7 (2.2-3.5) F 3.5 (2.4-4.3)
Current smokers, n (%)	T 101 (50.2) M 87 (62.1) F 14 (22.9)	T 42 (35.3) M 31 (52.5) F 11 (18.3)	T 61 (59.2) M 52 (68.4) F 9 (33.3)	T 40 (40.8) M 35 (54.7) F 5 (14.7)
Diabetes mellitus, n (%)	T 52 (25.9) M 30 (21.4) F 22 (36.1)	T 17 (14.3) M 8 (13.6) F 9 (15.0)	T 29 (28.2) M 18 (23.7) F 11 (40.7)	T 23 (23.5) M 12 (18.8) F 11 (32.4)
Arterial hypertension, n (%)	T 140 (69.7) M 88 (62.9) F 52 (85.2)	T 67 (56.3) M 29 (49.2) F 38 (63.3)	T 60 (58.3) M 39 (51.3) F 21 (77.7)	T 80 (81.6) M 49 (76.6) F 31 (91.2)

Observed frequencies of the rare alleles of Thr312Ala FGA, 10034 C > T FGG, Leu564Pro FXIII-A and Arg6Trp PI polymorphisms in the overall studied population were 20.5%, 17.3%, 14.3%, 19.7%, respectively. The Chi-square (χ^2^) test for the independence between allelic classes did not indicate a departure from the Hardy-Weinberg equilibrium in any study group (Table 2).

**Table 2:** Distribution of Thr312Ala FGA, 10034C > T FGG, Pro564Leu FXIII-A and Arg6Trp PI genotypes and alleles by coronary artery disease and myocardial infarction Data are presented as number (percentage) of participants. CAD, coronary artery disease; MI, myocardial infarction; HWE, Chi-square test and statistical significance of departure from the Hardy-Weinberg equilibrium.

	CAD+ (n=201)	CAD- (n=119)	CAD+ group only	
			MI+ (n=103)	MI- (n=98)
**Thr312Ala FGA**				
AA	128 (63.7)	74 (62.2)	64 (62.1)	64 (65.3)
AG	65 (32.3)	40 (33.6)	34 (33.0)	31 (31.6)
GG	8 (4.0)	5 (4.2)	5 (4.9)	3 (3.1)
A	321 (79.9)	188 (79.0)	162 (78.6)	159 (81.1)
G	81 (20.1)	50 (21.0)	44 (21.4)	37 (18.9)
HWE χ^2^; p-value	0.00; 0.944	0.02; 0.889	0.03; 0.860	0.11; 0.745
**10034C>T FGG**				
CC	146 (72.6)	76 (63.9)	75 (72.8)	71 (72.4)
CT	49 (24.4)	36 (30.3)	23 (22.3)	26 (26.5)
TT	6 (3.0)	7 (5.9)	5 (4.9)	1 (1.0)
C	341 (84.8)	188 (79.0)	173 (84.0)	168 (85.7)
T	61 (15.9)	50 (21.0)	33 (16.0)	28 (14.3)
HWE χ^2^; p-value	0.57; 0.452	0.93; 0.334	2.98; 0.084	0.68; 0.409
**Pro564Leu FXIII-A**				
TT	149 (74.1)	86 (72.3)	77 (74.8)	72 (73.5)
TC	48 (23.9)	30 (25.2)	24 (23.3)	24 (24.5)
CC	4 (2.0)	3 (2.5)	2 (1.9)	2 (2.0)
T	346 (86.1)	202 (84.9)	178 (86.4)	168 (85.7)
C	56 (13.9)	36 (15.1)	28 (13.6)	28 (14.3)
HWE χ^2^; p-value	0.00; 0.953	0.04; 0.843	0.01; 0.935	0.00; 0.999
**Arg6Trp PI**				
CC	138 (68.7)	72 (60.5)	69 (67.0)	69 (70.4)
CT	57 (28.4)	37 (31.1)	30 (29.1)	27 (27.6)
TT	6 (3.0)	10 (8.4)	4 (3.9)	2 (2.0)
C	333 (82.8)	181 (76.1)	168 (81.6)	165 (84.2)
T	69 (17.2)	57 (23.9)	38 (18.4)	31 (15.8)
HWE χ^2^; p-value	0.00; 0.969	2.55; 0.110	0.11; 0.746	0.12; 0.732

After the adjustment for age, gender, body mass index, current smoking, total cholesterol, triglycerides and HDL-cholesterol using the multivariable, binary logistic regression, 10034 C > T FGG and Arg6Trp PI CC genotypes were significantly associated with CAD (Table 3). Patients with 10034 C > T FGG CC genotype had 3.5 times (95% CI 1.02-12.03) higher odds for CAD than patients with 10034 C > T FGG TT genotype. Patients with Arg6Trp PI CC genotype had 3.86 times (95% CI1.23–12.12) higher odds for CAD than patients with Arg6Trp PI TT genotype. However, among the female subjects with aforementioned genotypes, higher odds for CAD were also observed but were not statistically significant, while among the male subjects they were (Table 3).

**Table 3:** Association of Thr312Ala FGA, 10034C > T FGG, Pro564Leu FXIII-A and Arg6Trp PI genotypes with coronary artery disease. Data are presented as number (percentage) of participants; 1 represents the referent category. CAD, coronary artery disease; OR, odds ratio for CAD; CI = confidence interval; p = statistical significance calculated using binary logistic regression. * Analysis was adjusted for age, gender (only for total), body mass index, current smoking, total cholesterol, triglycerides and HDL-cholesterol.

		n	Prevalence of CAD	Unadjusted	Multivariable, adjusted*	
				OR (95% CI)	OR (95% CI)	p
**Thr312Ala FGA**						
AA	total	202	128 (63.4)	1.08 (0.34-3.43)	1.08 (0.32-3.72)	0.898
	male	118	85 (72.0)	1.29 (0.30-5.45)	1.18 (0.24-5.73)	0.839
	female	84	43 (51.2)	1.05 (0.14-7.80)	0.75 (0.09-6.07)	0.791
AG	total	105	65 (61.9)	1.02 (0.31-3.32)	0.84 (0.23-2.98)	0.782
	male	72	49(68.1)	1.07 (0.25-4.64)	0.79 (0.15-3.94)	0.761
	female	33	16 (48.5)	0.94 (0.12-7.50)	0.75 (0.08-6.97)	0.798
GG	total	13	8 (61.5)	1	1	
	male	9	6 (66.2)	1	1	
	female	4	2(50.0)	1	1	
**10034C>T FGG**						
CC	total	222	146 (65.8)	2.24 (0.73-6.90)	3.50 (1.02-12.03)	0.047
	male	135	98 (72.6)	3.18 (0.92-11.05)	3.82 (0.97-15.04)	0.045
	female	87	48 (55.2)	1.23 (0.08-20.32)	2.20 (0.10-51.6)	0.798
CT	total	85	49 (57.6)	1.59 (0.49-5.13)	2.54 (0.71-9.16)	0.154
	male	53	37(69.8)	2.78 (0.74-10.42)	3.82 (0.89-16.47)	0.072
	female	32	12 (37.5)	0.60 (0.03-10.51)	1.03 (0.04-25.4)	0.984
TT	total	13	6 (46.2)	1	1	
	male	11	5 (45.5)	1	1	
	female	2	1(50.0)	1	1	
**Pro564Leu FXIII-A**						
TT	total	235	149 (63.4)	1.30 (0.28-5.94)	1.76 (0.35-8.81)	0.492
	male	151	104 (68.9)	0.74(0.08-7.28)	0.85 (0.08-9.71)	0.899
	female	84	45 (53.6)	2.31 (0.20-26.44)	3.81 (0.29-49.4)	0.307
TC	total	78	48 (61.5)	1.20 (0.25-5.74)	1.72 (0.32-9.19)	0.529
	male	44	33 (75.0)	1.00 (0.09-10.63)	0.96 (0.08-12.36)	0.977
	female	34	15 (44.1)	1.58 (0.13-19.12)	3.03 (0.21-42.88)	0.984
CC	total	7	4 (57.1)	1	1	
	male	4	3 (75.0)	1	1	
	female	3	1 (33.3)	1	1	
**Arg6Trp PI**						
CC	total	210	138 (65.7)	3.19 (1.12-9.14)	3.86 (1.23-12.12)	0.021
	male	128	97(75.8)	3.13 (0.85-11.52)	4.18 (1.03-16.92)	0.045
	female	82	41 (50.0)	5.00 (0.56-44.69)	3.81 (0.38-38.57)	0.257
CT	total	94	57 (60.6)	2.57 (0.86-7.66)	2.92 (0.89-9.58)	0.076
	male	61	38 (62.3)	1.65 (0.43-6.33)	2.21 (0.53-9.29)	0.278
	female	33	19 (57.6)	6.79 (0.71-64.73)	5.50 (0.51-59.61)	0.161
TT	total	16	6 (37.5)	1	1	
	male	10	5 (50.0)	1	1	
	female	6	1 (16.7)	1	1	

**Figure 2:**
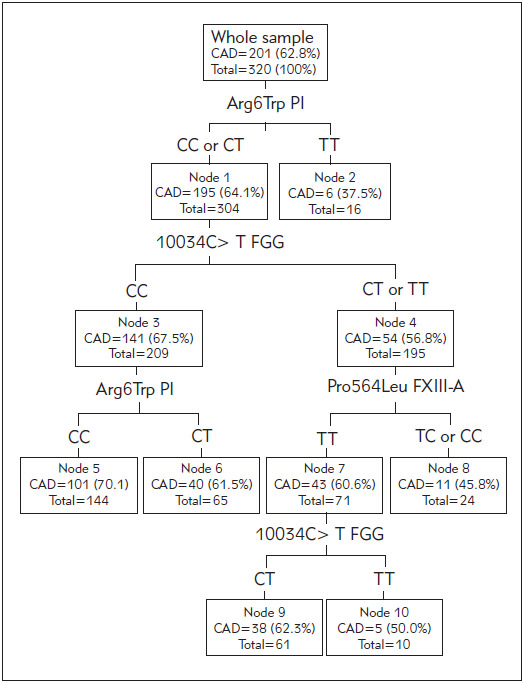
Classification tree calculated using a CART growing method with the cross-validation performed using 25 subsamples (n=320).

Using the classification tree analysis, we observed the highest prevalence of CAD (70.1%) in individuals with both Arg6Trp PI CC and 10034C > T FGG CC genotypes (node 5 in Figure 2). In our sample, there were 144 (45.0%; 95% CI 39.5%-50.6%) individuals with this »high-risk« genotype. Two segments of individuals had the »moderate« prevalence of CAD about the average of the total sample (nodes 9 and 6 in Figure 2) with the pooled prevalence of CAD 61.9%. These were the patients either with Arg6Trp PI CT and 10034C > T FGG CC genotypes (node 6 in Figure 2), or the patients with Arg6Trp PI CC or CT, and Pro564Leu FXIII-A TT, and 10034C > T FGG CT genotypes (node 9 in Figure 2). In our sample, there were 126 (39.4%; 95% CI 34.0%-45.0%) individuals with this, »medium-risk« genotypes. Three segments of individuals with the lowest prevalence of CAD (pooled estimate 44%) were those with Arg6Trp PI TT genotype (node 2 in Figure 2) or those with Arg6Trp PI CC or CT and 10034C > T FGG CT or TT and Pro564Leu FXIII-A TC or CC genotypes (node 8 in Figure 2) or the patients with Arg6Trp PI CC or CT and Pro564Leu FXIII-A TC or CC and 10034C > T FGG TT genotypes (node 10 in Figure 2). In our sample, there were 50 (15.6%; 95% CI 11.8%-20.0%) patients with these »lower risk« genotypes. After the adjustment for all covariates, the differences in the odds for CAD between these three »risk-segments« were significant. The »moderate-risk« segment had 2.37 (95% CI 1.15-4.92; p=0.020), and the »highestrisk« segment had 3.50 (95% CI 1.69-7.25; p=0.001) higher odds for CAD than the »lower-risk« segment.

We have not observed the significant association of any of the four polymorphisms with the history of MI (data not shown). The analysis of the potential association of investigated polymorphisms with fibrinogen levels showed that the CAD+ individuals with TT Pro564LeuFXIII-A genotype have statistically significant higher fibrinogen levels than CAD-individuals (2.80, 95% CI 2.30-3.70; p=0.017). No other association have been noticed (data not shown).

## Discussion

Since the first linkage/association studies and candidate-gene studies, through the first GWAS study of CAD in 2007 and up to current days when MEGASTROKE and CARDIoGRAMplusC4D consortia exist, scientists have been trying to combine data to elucidate the genetic variants that influence CAD development. Aiming to contribute to those efforts, we conducted this study on Croatian patients to investigate the potential association of four polymorphisms located in the genes coding for coagulation factors.

The first in the line of the key players in the process of clot formation is a molecule of fibrinogen. Two SNPs, located in the different polypeptide chains of fibrinogen, were in the focus of this study. The frequency of the less abundant form of Thr312Ala SNP (rs6050), located in the fibrinogen alpha chain (FGA), among screened individuals from Croatia, was 20.5%, which is in concordance with the frequency among the overall European population. The Ala312 frequency is known to vary between different populations – the Africans and Asians, for example, have this variant almost twice as often present in their population compared to the Europeans [23]. Neither the significant difference regarding the prevalence of the particular Thr312Ala FGA genotype between the CAD+ and CAD-individuals was observed in this study, nor this missense mutation showed association with the risk for developing CAD/MI. The literature data regarding this SNP and its functional consequences and disease-associations are quite contradictory. Lim and collaborators have found that firmer clots, with reduced permeability, occurred at higher fibrinogen concentrations in the presence of Thr312FGA and Val34 FXIII-A [24], while the Ala312 allele has been associated with thicker fibrin fibres and increased alpha-chain cross-linking by FXIIIa [16] [25]. According to our findings, there is no association of fibrinogen levels, Thr312Ala FGA and risk of CAD. Our findings are in concordance with the finding of Collet and colleagues [26], who have not found associations between Ala312 FGA variants and clots strength/porosity. Similarly, some studies showed no associations of Thr312Ala with ischemic stroke (IS) [27] or myocardial infarction [26], while others reported the association of this SNP with IS and MI [28], venous thromboembolism (VTE) [29] [30] [31], chronic thromboembolic pulmonary hyper tension [32], intracerebral haemorrhage [33], as well as for pregnancy loss and a hypercoagulable state in later pregnancy [34].

Another SNP in the fibrinogen gene, but this one in the gamma-chain gene (rs2066865, 10034 C > T FGG), has been associated with venous thromboembolism and was also investigated in this CAD-study. LETS researchers found an association of the 10034C > T FGG polymorphism and increased risk of VTE [35], and the association was confirmed by several other studies [36] [37] [38] [39] [40] [41], even in the context of higher risk for VT among cancer patients with specific FGG genotypes [42]. According to our results, this polymorphism is also associated with CAD. In our study, patients with 10034C > T FGG CC genotype had 3.5 times (95% CI 1.02-12.03) higher adjusted odds for CAD than patients with 10034C > T FGG TT genotype. The FGG allele frequencies in this cohort were somewhat different, but comparable with the frequencies for European population (T allele frequency 17.3% vs 22.1%, respectively), which is considerably lower than the frequencies in Africa (35.2%) and East Asia (44.1%) [43]. More intriguing, it should be emphasised that aforementioned association of rs2066865 and VT is confirmed only for Caucasian population, while the studies performed so far showed no association in the African-American [38], Chinese [44] or, according to the meta-analysis of Jiang and collaborators, non-white population [41]. This finding also highlights the importance of performing the same association studies on different populations. Though 10034C > T FGG is located in the 3'-untranslated region of the FGG gene, it is known that it reflects in the protein structure and consequently, functionality. The 10034C > T FGG SNP, located in a cleavage stimulatory factor sequence, may interfere with pre-mRNA cleavage [35]. Allele T favours the formation of alternatively spliced fibrinogen γ'-chain instead of g-chain [17], and fibrin containing γ'-variant binds thrombin more effectively, which may explain the reduced tendency to thrombosis among individuals with high levels of γ'-variant. This is the current hypothesis though the mechanism by which the rs2066865 affects susceptibility to VTE is not fully elucidated [45]. We can assume that the underlying mechanism in CAD patients is the same and that this explanation for 10034C > T FGG and VTE association is valid perhaps also for CAD.

The association of Pro564Leu FXIII-A (rs5982), which is assumed to affect the mutual binding of the FXIII-A and FXIII-B subunits and consequently contribute to change in FXIII activity, and CAD/MI was also investigated in this study. Pro564 variant seems to be associated with an increased level of fibrinogen in CAD patients, according to our study. That observation, though shown to be statistically significant, should be taken with caution, because the elevated levels of fibrinogen still fall into what is considered »a normal, reference interval«. Therefore, its biological significance is questionable and requires further investigation. The obtained prevalence of the Leu564 FXIII-A allele in Croatian patients was 14%, and no association between the presence of the Leu564 FXIII-A allele and risk for CAD or MI have been found. In clinical practice, it is well-known that, in spite of the documented presence of advanced CAD, only a subset of patients develops acute MI during their life-course. The reasons for individual differences in susceptibility to MI are poorly understood. Both CAD and MI are multifactorial disorders, in which a single factor contributes little to the susceptibility to the disease. This contribution can also be masked with the influence of other, known and mostly still unknown factors and therefore, this contribution is sometimes hard to be noticed. This polymorphism was previously associated with an increased risk of intracerebral haemorrhage in women younger than 45 years by Reiner et al. [46]. Still, Pruissen and colleagues did not find any association between Pro564Leu FXIII and ischemic stroke [47]. Assuming a dominant inheritance of Leu564 variant of FXIII-A, Siegernik and colleagues linked it also with increased risk of IS in women (OR= 1.40; 95% CI 1.01 to 1.93), but due to fact that the increased risk was found in heterozygotes, and there was no response in homozygotes, authors concluded that there are no satisfactory answer and biological explanations for this situation [8]. Regarding the rs2070863 (C > T), which causes amino acid substitution Arg6Trp PI, the observed frequency of an Arg6PI and Trp6PI allele in our subjects were found to be 80.0% and 20.0%, respectively, and it is consistent with the data published by previous studies [19] [48]. More importantly, the patients with Arg6Trp PI CC genotype had 3.86 times (95% CI 1.23-12.12) higher odds for CAD than the patients with Arg6Trp PI TT genotype. Christiansen and collaborators suggested a role of Arg6TrpPI polymorphism in governing the long-term deposition or removal of intravascular fibrin through the exchange of Met-PI/Asn-PI ratio that affects the rate of PI incorporation into fibrin. The Met-PI of the Trp6PI homozygous carriers is less susceptible to the cleavage by APCE, and consequently, less Asn-PI is available for the efficient incorporation into forming fibrin which becomes more susceptible to the digestion by plasmin [19]. Therefore, it could be expected that the presence of the Trp6PI allele could be protective in AT [49].

It should also be mentioned that additional splitting into subgroups according to gender revealed that observed higher odds regarding both 10034C > T FGG and Arg6Trp PI CC genotypes are gender-related, and the only combination of those genotypes and male gender seems to increase susceptibility for CAD, while female gender seems to eliminate that effect. It is reasonable to assume that there is some physiological background responsible for that outcome, and we can hope that some other study will give us more insight into it.

The classification three analysis of the results presented in our study revealed the highest prevalence of CAD in the patients with the combinations of particular FGG and PI genotypes, namely CC/CC, which can be considered »high-risk« genotypes when taking into consideration that 70% of individuals involved in this study that bear this genotype were individuals with confirmed CAD.

The main limitation of this research is its retrospective nature and quite a moderate number of individuals in the studied groups. Additionally, we selected the consecutive and not the random sample from the targeted population. Therefore, it is possible that our sample is somewhat biased toward the subpopulation with more pronounced subjective symptoms. Moreover, there is an imbalance in gender distribution due to the process of patient selection. The study subjects were recruited from the patients consecutively referred to the hospital for coronary angiography and suspected coronary artery disease. According to the published studies, there is a marked difference in coronary artery disease severity and burden between the females and males presenting for the investigation of suspected angina. Male sex is a well-established risk factor for cardiovascular disease, whereas women are more likely to have normal coronary arteries or less severe disease than age-matched men. Thus, as it was planned to recruit approximately 350 subjects in the study by the consecutive selection, more male subjects entered the study. Furthermore, for the control group, we used patients with negative findings on angiography. It can be argued that those individuals cannot be considered truly healthy because they had risk factors that led to their angiography in the first place. However, we believe that this makes our data stronger because the differences observed might have been much greater if a control group of patients without any risk factors have been used. The advantage of this study is accurate phenotyping by coronary angiography, which allowed us a comparison of patients with angiographically proven, advanced CAD, with or without MI.

Up to now, candidate-gene analysis studies, as well as GWAS studies, pointed to numerous loci possible associated with the risk of CAD or MI. However, a meta-analysis of the available data identified only around 60 common variants convincingly associated with coronary artery disease, some in the Europeans [50] [51], other in both European and non-Caucasian populations [52] [53], which is not a significant number considering the fact that estimated heritability of CAD range from 30 up to 60% [54]. It is also important to mention that the majority of these loci show pleiotropy, meaning that they are also associated with other diseases or traits. Additionally, new investigations sometimes reveal novel susceptibility genes, which require additional validation before firm vascular biology links can be established. The structure of fibrin clot and the effect of coagulation factors' genetic variants on its cross-linking are very complex. So far, we can conclude that the genetic susceptibility to CAD is largely determined by common SNPs of small effect size and that not all of them are at the moment known to us. Considering that the impact of genes could be diverse in various environments and consequently, the risk of the disease could be different, to our knowledge, this is the first investigation of the association of Thr312Ala FGA, 10034C > T FGG, Pro564Leu FXIII-A and Arg6Trp PI polymorphisms with CAD in the group of Croatian patients.

## Conclusion

Our finding suggests that 10034C > T FGG and Arg6Trp PI are associated with CAD, and CC genotypes for both SNPs represent a risk factor for CAD.

## Funding

This work was supported by grant #006-0061194-1218 and grant #108-1080316-02 from the Ministry of Science, Education and Sports of the Republic of Croatia.

## Acknowledgements

We are grateful to the patients for their participation in this study

## Conflict of interest statement

The authors state that they have no conflicts of interest regarding the publication of this article.
